# Necrotic scleral melt and fungal keratitis—a complication of subtenon triamcinolone acetonide injection

**DOI:** 10.1186/s12348-020-0197-4

**Published:** 2020-01-27

**Authors:** Manisha Agarwal, Richa Ranjan, Umang Mathur

**Affiliations:** grid.440313.1 Dr. Shroff’s Charity Eye Hospital, 5027-Kedar Nath Road, Daryaganj, New Delhi, 110002 India

**Keywords:** Subtenon triamcinolone acetonide injection, Necrotic scleral melt, non-necrotizing, non-infectious anterior scleritis, High myopia

## Abstract

**Purpose:**

Subtenon triamcinolone acetonide injection (STAI) is a safe drug delivery method for various ocular conditions. We report two cases of necrotic scleral melt, a rare complication of STAI.

**Methods:**

The first patient received STAI for post-operative inflammation control and developed necrotic scleral melt at the site of STAI with superadded fungal keratitis.

The second patient received three STAI for non-necrotizing, non-infectious anterior scleritis and developed scleral necrosis at the site of her last STAI. Noncompliance with medications resulted in the progression of scleral necrosis to a new area.

**Results:**

In the first patient, surgical removal of triamcinolone deposit resulted in healing of the scleral melt while the second patient was managed conservatively with corticosteroids and immunosuppressants.

**Conclusion:**

Scleral melt is a rare complication of STAI; however, an early diagnosis and management of any predisposing factor along with surgical debridement should be considered as a potential critical treatment option to salvage the eye.

## Introduction

Periocular steroid injection (PSI) is often used after intraocular surgery and various inflammatory ocular diseases. This drug delivery method provides prolonged drug activity with minimal systemic side effects; however, ocular side effects include development of cataract and glaucoma [1]. Conjunctival necrosis and scleritis though reported are rare complications of subtenon triamcinolone acetonide injection (STAI) [2, 3]. We report two cases of scleral necrosis and melt following STAI given for management of post-operative inflammation in one patient and non-necrotizing, non-infectious anterior scleritis (NNAS) in the second patient with underlying granulomatosis with polyangiitis.

## Case 1

A 62-year-old female presented with redness in the right eye (RE) for the last 1 month. She gave a history of high myopia and vitreoretinal surgery done elsewhere for rhegmatogenous retinal detachment with macular hole in the RE 1 month back. The surgery done was pars plana vitrectomy with internal limiting membrane peeling with silicone oil injection, encirclage band was not used. At the end of the surgery, STAI (0.5 cc of triamcinolone acetonide suspension, 40 mg/ml) was injected in the subtenon’s space in the superonasal quadrant (SNQ) to control post-operative inflammation.

**Fig. 1:**
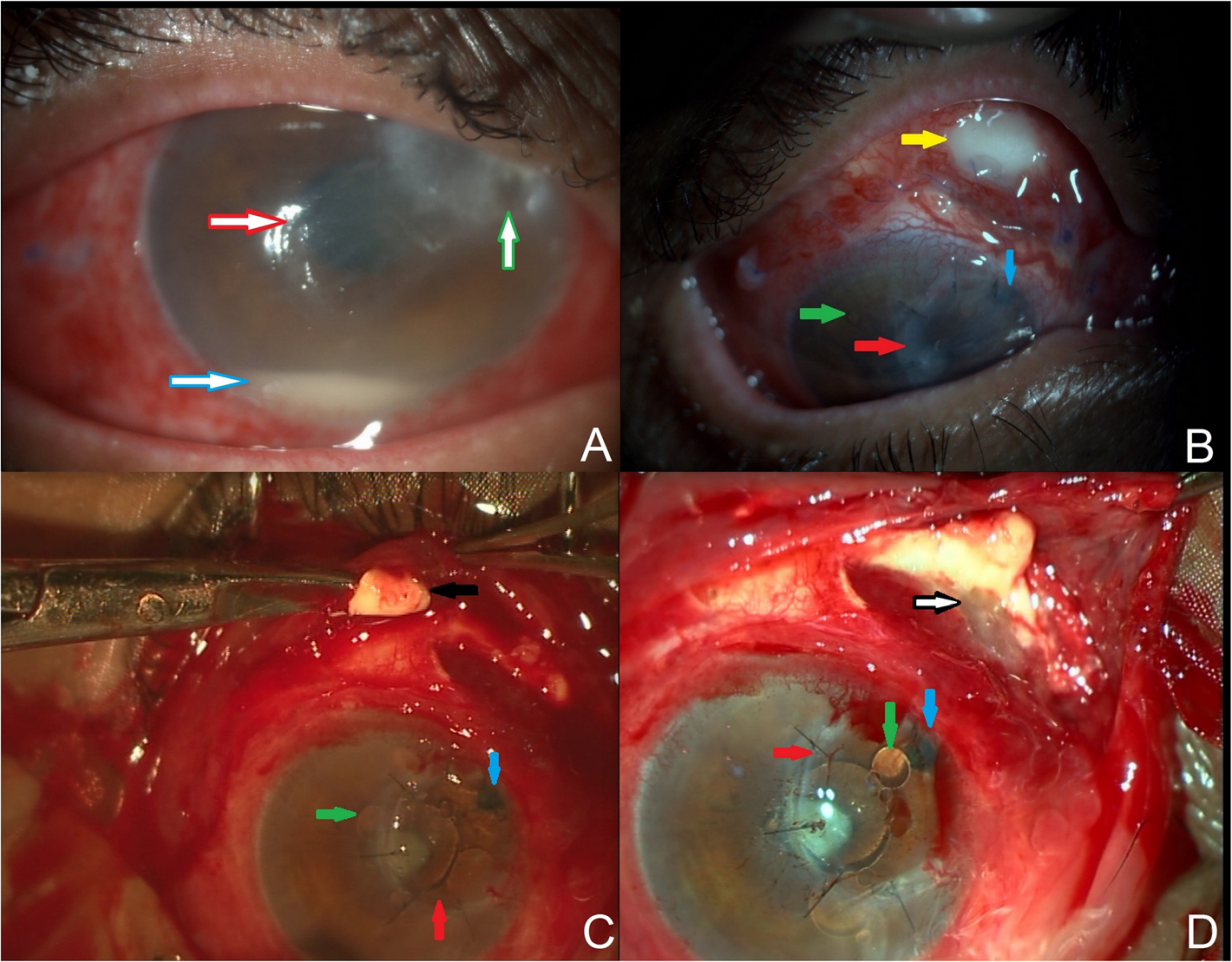
Anterior segment photo of the right eye showing **a** circumcorneal congestion, epithelial defect (red border arrow), stromal infiltration with corneal thinning (green border arrow) and a hypopyon (blue border arrow). **b** Corneal patch graft (red arrow), silicone oil bubbles (green arrow) in the anterior chamber, surgical PI (blue arrow) and scleral abscess (yellow arrow) in the superonasal quadrant. **c**, **d** Intraoperative photo of the right eye showing corneal patch graft (red arrow), silicone oil bubbles (green arrow), surgical PI (blue arrow), dissection of the sub-tenon triamcinolone deposit (black arrow) and extensive scleral melt (black border arrow)

On examination, the best-corrected visual acuity (BCVA) in the RE was finger counting (FC) half meter, <N36 and in the left eye (LE) 6/36, N12. Intraocular pressure (IOP) was 16 mmHg in the LE and was deferred in the RE. The anterior segment of the RE showed circumcorneal congestion, central epithelial defect (3 × 4 mm), corneal thinning with stromal infiltration (3 × 4 mm) in the SNQ and hypopyon (2 mm) with a cataractous lens (Fig. 1a). The LE was normal. The fundus examination of the RE had a hazy view and the LE showed myopic fundus with posterior staphyloma.

Microbiological smear examination of the corneal scraping was positive for fungus. The patient was started on topical voriconazole 1%, natamycin 1%, moxifloxacin 0.5%, and lubricating eye drops. Her investigations were negative for any underlying collagen vascular disease.

Follow-up at 1 week, as there was no improvement, she underwent a therapeutic corneal patch graft (CPG) with intracameral voriconazole injection (IVI) (50 μg/0.1 ml). The culture of the corneal button showed Aspergillus flavus. Oral ketoconazole 200 mg twice a day was added.

Follow-up at 3 weeks, BCVA in the RE was hand movement, <N36. The anterior segment showed an abscess in the SNQ of the sclera, a well apposed CPG in the SNQ and silicone oil bubbles in the anterior chamber (AC) with peripheral iridectomy (Fig. 1b). She underwent a repeat surgery during which subtenon triamcinolone deposit was dissected out from the SNQ and underlying extensive scleral melt with necrosis was noted (Fig. 1c, d). The suppurative material was removed and the conjunctiva was closed after a thorough povidone-iodine 5% wash with a repeat IVI (50 μg/0.1 ml). The culture of the necrotic scleral tissue showed no growth. Post-operatively, she was symptomatically better and the scleral melt showed gradual healing.

**Fig. 2:**
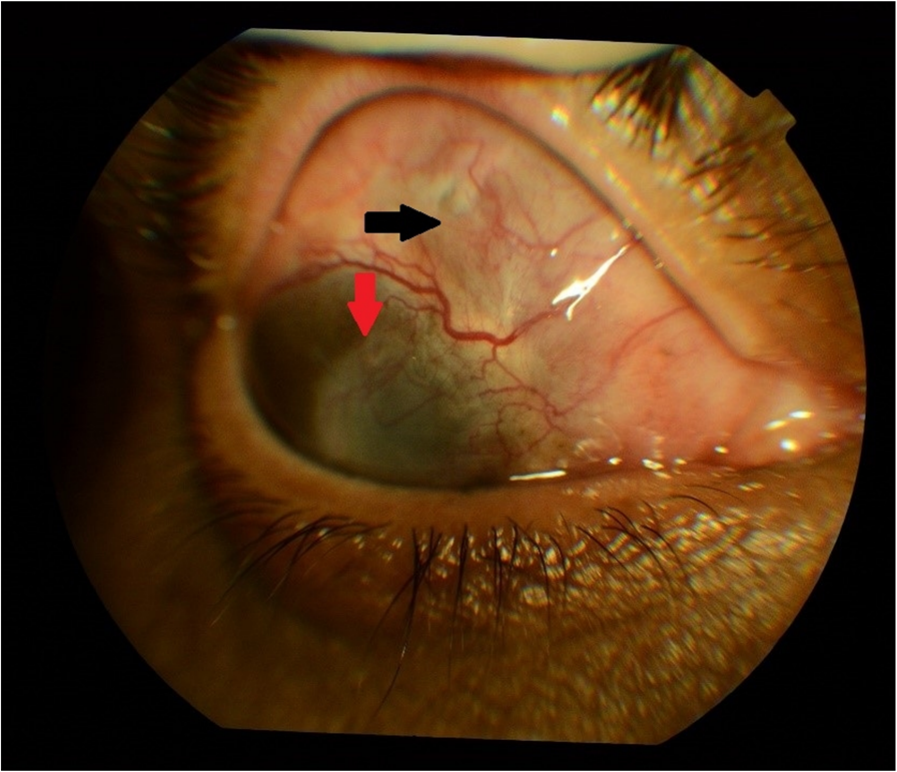
Anterior segment photo of the right eye showing leucomatous corneal opacity with vascularized cornea (red arrow) and healed scleral melt in the superonasal quadrant of the sclera (black arrow)

Nine months follow-up, BCVA in the RE was hand movement, <N36. The anterior segment showed a leucomatous corneal opacity with vascularized CPG and complete healing of the scleral melt (Fig. 2).

## Case 2

A 46-year-old female presented with recurrent redness and pain in the RE for the last 4 years. Past history included treatment done elsewhere with oral and topical steroids along with three STAI (subtenons injection of triamcinolone acetonide suspension, 40 mg/ml) in the RE given in the last 3 years for NNAS with the last injection given 1 week back following which she had an increase in redness and pain. She was a known patient of hypertension and granulomatosis with polyangiitis on oral methotrexate 10 mg once a week, oral folic acid 5 mg and anti-hypertensive medications for the last 3 years.

**Fig. 3:**
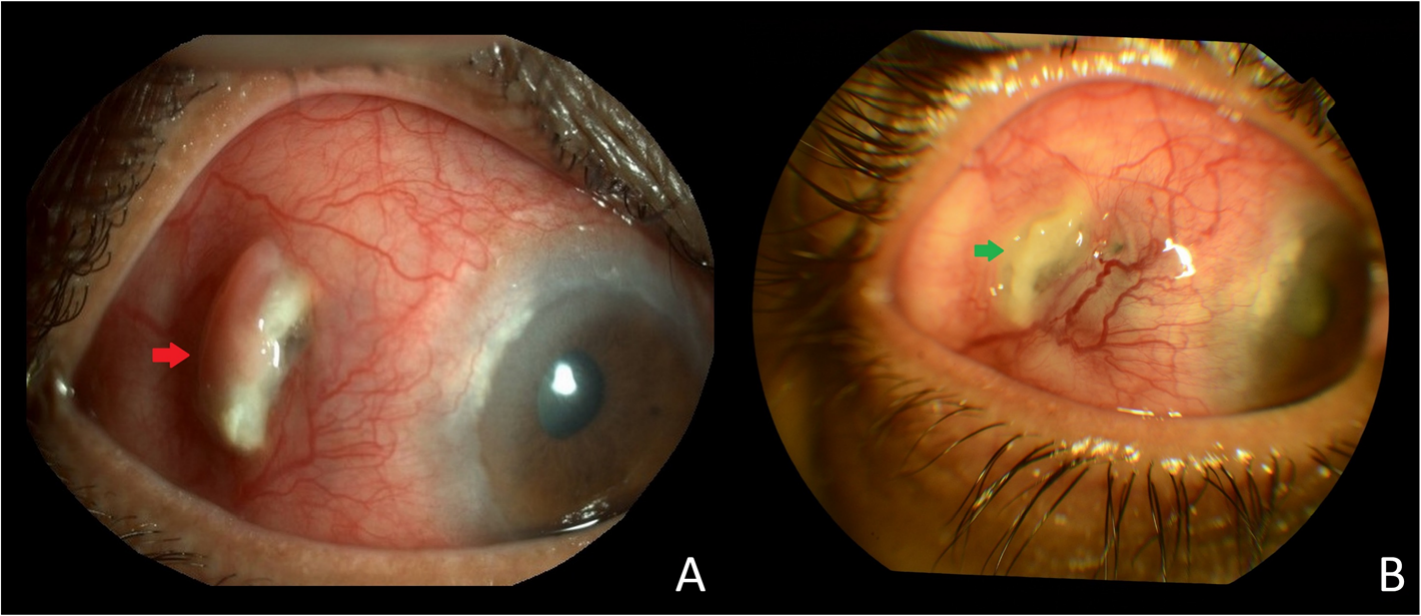
Anterior segment photo of the right eye showing **a** episcleral and scleral congestion along with scleral thinning and a nodule formation in the temporal region (red arrow). **b** Healing of the area of necrotic scleral melt (green arrow)

On examination, the BCVA in the RE was FC 1 m, <N36 and in the LE was 6/18,N18. IOP was 10 mmHg in both eyes. The anterior segment of the RE showed episcleral and scleral congestion along with scleral thinning with a nodule formation in the temporal region (Fig. 3a), posterior synechiae and complicated cataract with no AC cells. The LE was normal. The fundus examination of the RE had a hazy view due to cataract and the LE was normal. B-scan showed a well-attached retina in the RE.

Her investigations showed raised cytoplasmic antineutrophil cytoplasmic antibody (C-ANCA-232.15 U/ml), erythrocyte sedimentation rate (ESR-51 mm/h) and C-reactive protein (CRP-8.36 mg/L); however, her perinuclear antineutrophil cytoplasmic antibody (P-ANCA-2.52 U/ml) levels were normal.

A diagnosis of necrotizing scleritis with scleral melt secondary to STAI was made and treatment started with topical prednisolone acetate 1%, atropine 1%, oral methotrexate 20 mg once a week and oral folic acid 5 mg. One month follow-up showed healing of the scleral melt (Fig. 3b).

**Fig. 4:**
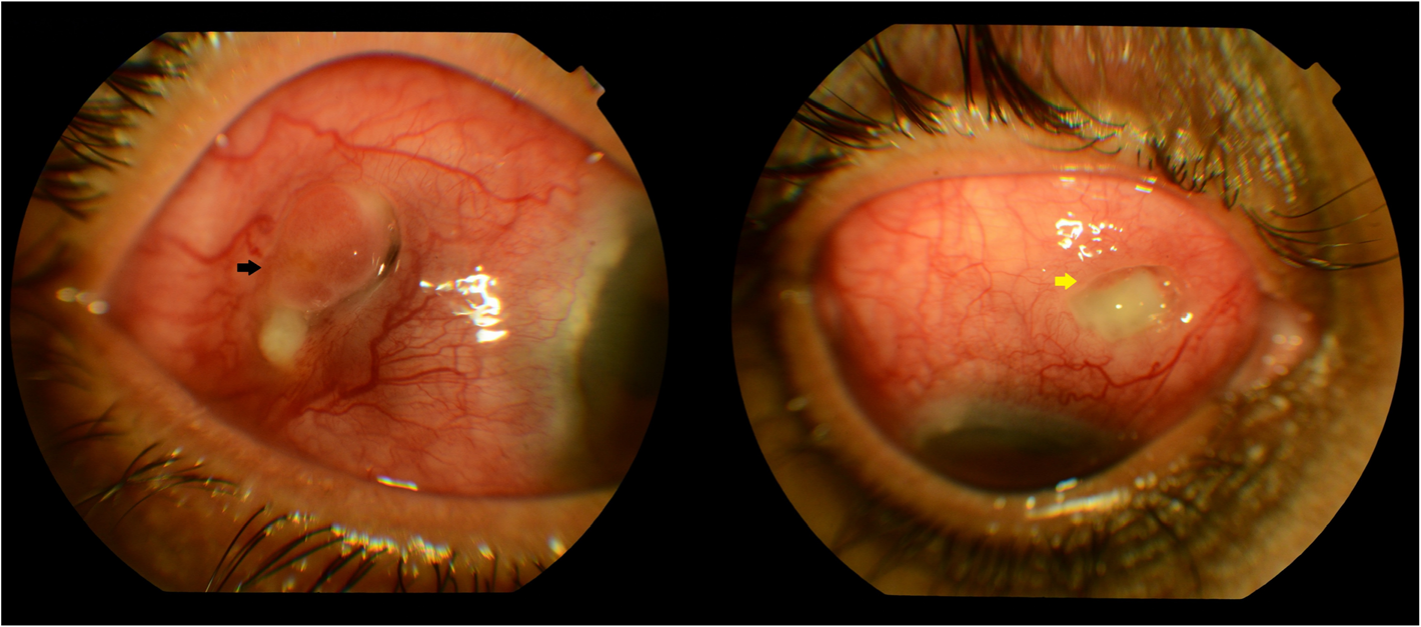
Anterior segment photo of the right eye showing marked episcleral and scleral congestion with the area of necrotizing scleritis and scleral melt in the temporal quadrant (black arrow) and a new area of scleral melt in the superonasal quadrant (yellow arrow)

However, she was lost to follow up for 4 months and had stopped her medications. She presented again with BCVA of FC 1 m, <N36 in the RE. The anterior segment of the RE showed marked episcleral and scleral congestion with an area of necrotizing scleritis and scleral melt in the temporal quadrant and a new area in the SNQ (Fig. 4). Her C-ANCA (171.96 U/ml), CRP (3.93 mg/l), and ESR (45 mm/h) were raised; however, P-ANCA (0.53 IU/ml) levels were normal.

She was started on topical prednisolone acetate 1%, moxifloxacin 0.5%, high-dose oral corticosteroids (1.5 mg/kg wt. and tapered) and immunosuppressants (cyclophosphamide 100 mg once a day) by a rheumatologist after systemic evaluation. She was on regular follow-up. Four months follow-up showed healing of both the areas of the scleral melt and the patient was symptomatically better.

## Discussion

Corticosteroids are used for various inflammatory ocular pathologies. Oral corticosteroids are known to have several systemic side effects; however, PSI including STAI have recently become popular due to their prolonged drug release and reduced systemic side effects. However, PSI has ocular complications such as IOP elevation and cataract formation [1], other rare complications include conjunctival necrosis and ischemia [2, 3].

There are reports on the usage of PSI for NNAS [4, 5]; however, STAI for managing scleritis is controversial due to the alleged risk of scleral necrosis and melt.

Sohn EH et al. [4], in their long-term multicenter retrospective series reported a good response with sub-conjunctival injection of 2–40 mg of triamcinolone acetonide with a mean of 2 triamcinolone acetonide injections per eye, in 68 eyes of NNAS with no case developing scleral melt or necrosis.

Autoimmune diseases are a potential risk factor for scleritis. Our second patient had granulomatosis with polyangiitis as an underlying cause of scleritis. She received 3 STAIs in the past 3 years for NNAS. However, she developed scleral necrosis and melt at the site of her last STAI, which may be due to the progression of the disease itself or triggered by the three STAIs which she received. Repeated PSI should be avoided in patients with an underlying pathology such as granulomatosis with polyangiitis as it may act as a trigger for the development of necrotizing scleritis. Management with immunosuppressants either in the form of increasing the dose of the drug or changing the immunosuppressant took care of both the scleritis and the underlying disease process.

Our first patient had no underlying systemic cause. She was a high myopic with associated thin sclera. She received a STAI at the end of her vitreoretinal surgery to control post-operative inflammation. However, she developed scleral necrosis and melt at the site of injection with superadded fungal keratitis. Scleral inflammation and necrosis can complicate the post-operative course of ocular surgery and can lead to sight-threatening complications [6]. It is important to differentiate from necrotizing sclerokeratitis as it is an immune-mediated ocular inflammatory process, characterized by profound inflammation of the sclera and episclera along with peripheral ulcerative keratitis, with presence of a potentially lethal systemic vasculitic process [7]. Necrotizing scleritis can be idiopathic, systemic disease-associated, infectious and associated with traumatic events and/or surgical procedures [8]. Das S et al. [6] have reported 4 cases of post-operative necrotizing scleritis with superadded infection in 2 of the cases. In their case series, treatment of the infection did not help in healing the area of necrotizing scleritis and immunosuppressants were needed to treat the scleral necrosis. Surgically induced necrotizing scleritis may be a result of a hypersensitivity reaction against an antigen revealed or altered due to multiple surgeries [9–11]. For appropriate management of post-operative scleritis, it is imperative to rule out infectious etiology first and immunosuppressives can be considered in non-responsive cases where infectious etiology has been ruled out [6]. Our first patient had undergone a single surgery and her investigations were negative for any underlying systemic cause. The treatment of the infection with broad-spectrum antibiotics and anti-fungals along with surgical removal of the triamcinolone deposit with CPG resulted in healing of the scleral necrosis and melt, without the use of any immunosuppressants. She did not have any recurrence in the follow-up visits. Therefore, we inferred that our first patient was not a case of necrotizing sclerokeratitis and developed scleral necrosis and melt secondary to triamcinolone acetonide injection possibly due to a thin sclera. There was a superadded fungal infection causing fungal keratitis of the persistent corneal epithelial defect, which may have been scraped during the surgery for better visualization.

Eslampour A et al. [3] have reported a case of conjunctival necrosis with scleritis and Staphylococcus saprophyticus infection following a STAI after vitrectomy for a metallic intraocular foreign body removal in a young boy. They too excised the remnants of triamcinolone which resulted in healing along with broad-spectrum antibiotics. However, they hypothesized that the sub-conjunctival spread of the STAI maybe the possible cause.

To the best of our knowledge, this is the first report of scleral necrosis and melt following STAI though conjunctival necrosis has been reported in the past. We report two cases of scleral necrosis and melt following STAI for the control of post-operative inflammation and for NNAS secondary to granulomatosis with polyangiitis to highlight the fact that though a rare complication of STAI but may occur with or without any underlying cause. We need to be aware of this complication while giving STAI; however, early diagnosis and management of any predisposing factor along with surgical debridement should be considered as a potential critical treatment option to salvage the eye.

## Data Availability

NA

## Abbreviations

AC: Anterior chamber
BCVA: Best-corrected visual acuity
C-ANCA: Cytoplasmic antineutrophil cytoplasmic antibody
CPG: Corneal patch graft
CRP: C-reactive protein
ESR: Erythrocyte sedimentation rate
FC: Finger counting
IOP: Intraocular pressure
IVI: Intracameral voriconazole injection
LE: Left eye
NNAS: Non-necrotizing, non-infectious anterior scleritis
P-ANCA: Perinuclear antineutrophil cytoplasmic antibody
PSI: Periocular steroid injection
RE: Right eye
SNQ: Superonasal quadrant
STAI: Subtenon triamcinolone acetonide injection

## References

[1] Kuo HK, Lai IC, Fang PC, Teng MC (2005). Ocular complications after a sub-tenon injection of triamcinolone Acetonide for uveitis. Chang Gung Med J.

[2] Jiun CY, Kuen WC, Shatriah I (2015). Conjunctival necrosis following a subconjunctival injection of triamcinolone Acetonide in a child. Middle East Afr J Ophthalmol.

[3] Eslampour A, Abrishami M, Tafaghodi S (2013). Conjunctival necrosis and scleritis following subtenon triamcinolone acetonide injection. Iran Red Crescent Med J.

[4] Sohn EH, Wang R, Read R (2011). Long-term, multi-center evaluation of subconjunctival injection of triamcinolone for non-necrotizing, non-infectious anterior scleritis. Ophthalmology.

[5] Zamir E, Read RW, Smith RE, Wang RC, Rao NA (2002). A prospective evaluation of subconjunctival injection of triamcinolone acetonide for resistant anterior scleritis. Ophthalmology.

[6] Das S, Saurabh K, Biswas J (2014). Postoperative necrotizing scleritis: a report of four cases. Middle East Afr J Ophthalmol.

[7] Hernández-Camarena JC, Rodríguez-García A, Valdez-García J (2015). Diagnosis and treatment approach for necrotizing scleritis (NS): a clinical case. Gac Med Mex.

[8] Sainz de la Maza M, Jabbur NS, Foster CS (1994). Severity of scleritis and episcleritis. Ophthalmology.

[9] Riono WP, Hidayat AA, Rao NA (1999). Scleritis: a clinicopathologic study of 55 cases. Ophthalmology.

[10] Fong LP, Sainz de la Maza M, Rice BA, Kupferman AE, Foster CS (1991). Immunopathology of scleritis. Ophthalmology.

[11] Grindle CF, Marshall J, McLeod D, Clarke E, Fison LG (1979). Complications of explants used in retinal detachment surgery. Mod Probl Ophthalmol.

